# Comparative and Adaptive Analyses of the Complete Chloroplast Genome Diversity in *Sium serra*

**DOI:** 10.3390/genes15121567

**Published:** 2024-12-03

**Authors:** SeongJun Park, SeonJoo Park

**Affiliations:** Department of Life Sciences, Yeungnam University, Gyeongsan 38541, Gyeongsangbuk-do, Republic of Korea; bsj1774@ynu.ac.kr

**Keywords:** *Sium serra*, chloroplast genome, substitution rate, adaptive evolution, phylogeny

## Abstract

Background/Objectives: *Sium serra* is distributed in Korea, China, and Japan. It was first identified as the genus *Pimpinella* and then reclassified as *Sium* by Kitagawa. Some *Sium* species are used as herbal medicine and are often confused with the similar form *Ligusticum sinense*. In this study, we analyzed the cp genome of *S. serra* and conducted comparative analyses with the cp genomes of related taxa. Methods: We extracted gDNA from fresh leaves and sequenced it using Illumina HiSeq2500. For the chloroplast genome assembly, de novo assembly was performed using Velvet v1.2.07. For the annotation, GeSeq and NCBI BLASTN were used. Afterwards, related taxa were analyzed using programs such as DnaSP and MISA. Results: *S. serra* was excluded from the study on the chloroplast (cp) genome in *Sium* because it was classified as *Pimpinella* in China. Therefore, this study aimed to analyze the cp genome of *S. serra* for the first time and its location within the genus *Sium*. The complete cp genome of *S. serra* was 154,755 bp in length, including a pair of inverted repeats, each 26,255 bp, a large single-copy region of 84,581 bp, and a small single-copy region of 17,664 bp. The cp genome comprised 79 protein-coding, 30 tRNA, and 4 rRNA genes. Furthermore, six regions of high nucleotide diversity were identified in the genus *Sium*. In the genus *Sium*, 1630 repeats that can serve as markers were also identified. Eight protein-coding genes with high K_A_/K_S_ values were under positive selection in the *Sium*. Our phylogenetic analyses suggest that *S. serra* was positioned with high bootstrap support within the *Sium* of the tribe *Oenantheae*, specifically in the southern Palearctic subclade. Conclusions: In this study, the *S. serra* chloroplast genome was sequenced and assembled. The genus *Sium* formed a monophyletic group; however, as not all the *Sium* species were included in this study, further research is necessary. This study can serve as foundational data not only for *Sium* but also for the tribe Oenantheae.

## 1. Introduction

According to the APG Ⅳ classification system, the genus *Sium* L. is classified within the tribe Oenantheae, in the family Apiaceae and subfamily Apioideae [1]. There are approximately 11 taxa of the genus *Sium*, which is primarily native to Eurasia and North America. *Sium* is a perennial herb that thrives in shady and wet environments [2,3]. *Sium* is characterized by its hairless appearance and its leaves, which are trifoliate or pinnate. In addition, it features compound inflorescences, and its fruit is ovate with distinct primary ribs and milk ducts [4,5]. The genus *Sium* is closely related to the genera *Berula* W.D.J. Koch and *Pimpinella* L. Some species of *Sium* have been reclassified into the genus *Berula* (*B. burchellii*, *B. bracteata*, *B. repanda*) [6,7]. *Pimpinella serra* has been reclassified into the genus *Sium*. However, in *Flora of China*, *S*. *serra* is still recognized as a synonym for *Pimpinella serra* [5].

In East Asia, some *Sium* species are employed in herbal medicine, primarily using their roots. These species are commonly used to treat headaches and high blood pressure. They contain numerous essential oils and chemical compounds. However, their dried roots are similar to those of “Chinese Gao ben” (*Ligusticum sinense*) and are often mistaken for and used interchangeably with it [8].

*S*. *serra* (Franch. & Sav.) Kitag. is known to be distributed in Korea, China, and Japan [4,5,9]. This species was first identified as *P*. *serra* by Franchet and Savatier in 1876 [10]. In 1941, Kitagawa reclassified the species into the genus *Sium* [11]. In Korea, it was first discovered by Lee and Ko in 2009 and named *S. ternifoilum* [12]. Later, in 2014, Chang renamed it *S. serrum,* which is now recognized as *S. serra* [13].

Plants, unlike other organisms, possess organelles called chloroplasts, which are responsible for photosynthesis and various metabolic processes [14,15]. The chloroplast (cp) genome, typically inherited maternally, ranges in size from 120 to 180 kb. The cp genome exists in a circular form and is divided between large single copy (LSC) and small single copy (SSC) by inverted repeats (IRs), which usually occur in two copies [16,17,18]. The cp genome contains approximately 79 protein-coding, 30 transfer RNA, and 4 ribosomal RNA genes [19]. This genome has a relatively slow mutation rate and high polymorphism between species, making it widely used in plant genetic identification and phylogenetic analysis [20].

In this study, the complete chloroplast genome of *S. serra* was constructed and annotated through de novo next-generation sequencing (NGS) and assembly. Therefore, this study aims to examine the complexity of the *Sium* cp genome structure, including the gene content, repeats, nucleotide diversity, and adaptive evolution.

## 2. Materials and Methods

### 2.1. Genomic DNA Isolation and Genome Sequencing

Fresh leaves of *S. serra* were collected from Wonju-Si, Gangwon State, Republic of Korea. Genomic DNA was isolated from the leaves using the Exgene Plant SV kit (GeneAll Biotechnology Inc., Seoul, Republic of Korea). The quality of the gDNA was measured using a Nano-200 spectrophotometer (Hangzhou Allsheng Instruments Inc., Hangzhou, China), and the quantity was verified using 1% agarose gel. NGS was conducted using the Illumina HiSeq2500, provided by Phyzen Ltd., Seongnam, Republic of Korea. The paired-end library (2 × 150 bp) was constructed using the Illumina platform. Sequencing was performed on paired reads with a 550 bp insert size, resulting in ~3 GB of raw data. Low-quality reads were identified using FastQC v0.11 and subsequently filtered out with Trimmomatic 0.39 [21,22].

### 2.2. Assembly and Annotation of the S. serra Chloroplast Genome

For the de novo cp genome assembly, plastid-like reads were extracted from the clean reads using the Velvet v1.2.07 [23]. The cp genome sequence was annotated using the GeSeq tool with NCBI BLASTN searches [24]. The architecture of the *S. serra* cp genome was visualized using the OGDRAW program [25].

### 2.3. Chloroplast Genome Sequence Divergence and Comparison

The chloroplast genome of *S. serra*, newly sequenced in this study, was compared with 6 publicly available *Sium* cp genomes (Supplementary Table S1) to determine the synteny of the cp genome structures. The IR boundary comparison diagram was manually created based on their plastome structures to access the expansion and contraction of the LSC, SSC, and IR junctions in the 7 *Sium* plastomes. The complete plastome sequences of all the *Sium* were analyzed for sequence similarity using mVISTA in Shuffle-LAGAN mode with default parameters, using the *S. serra* cp genome as a reference [26].

### 2.4. Genetic Divergence

The genetic divergence of the seven *Sium* cp genomes was calculated using the nucleotide diversity (Pi) to identify highly variable sites with DnaSP v6.12.03 [27]. The cp genomes were aligned using MAFFT implemented in Geneious Prime (Biomatters, Auckland, New Zealand) [28,29]. In the nucleotide diversity analysis, the window length was set to 800 bp and the step size to 200 bp using the DnaSP v6.12.03 [27].

### 2.5. Repeat Sequence

The simple sequence repeats (SSRs) motifs in the seven *Sium* plastomes were analyzed using MISA v2.1, with the minimum number of repeats thresholds set to ten repetitions for mononucleotide SSRs, six for dinucleotide SSRs, and five for tri-, tetra-, penta-, and hexanucleotide SSRs [30]. Tandem repeats were identified using the Phobos Tandem Repeats Finder v1.0.6 with parameters set to 1 for the matches, −5 for mismatches and gaps, and 0 for N positions [31]. Additionally, the forward, reverse, complement, and palindromic repeats were detected using REPuter with a Hamming distance of 3, minimum sequence identity of 90%, and minimum repeat size of 30 bp [32]. A single copy of the IR region was used for all these analyses.

### 2.6. Substitution Rate

The complete cp genome of *S. serra* was compared with those of six *Sium* species. The nonsynonymous (K_A_) and synonymous (K_S_) substitution rates were analyzed by extracting DNA sequences of the identical specific 79 functional protein-coding genes, translating them into amino acids, and aligning them independently using Geneious Prime (Biomatters, New Zealand). The K_A_ and K_S_ substitution rates were calculated using DnaSP v6.12.03 [27].

### 2.7. Phylogenetic Tree

Thirty-one cp genomes from Apioideae were selected to construct a phylogenetic tree, with four Araliaceae species chosen as an outgroup (Supplementary Table S1). This approach was used to determine the position of *S. serra* within Apioideae and analyze the phylogenetic relationship of the *Sium* genus. The cp genome sequences of 34 species from the Apiaceae and Araliaceae families were downloaded. The 79 protein-coding genes shared by 35 cp genomes were concatenated, aligned, and saved in FASTA format using MAFFT. The maximum likelihood (ML) tree was constructed using RAxML v7.2.6 with the general time reversible + γ + invariant model. One thousand nonparametric bootstrap replicates were performed to estimate the support for each internal branch of the phylogeny [33].

## 3. Results

### 3.1. General Features of the S. serra Chloroplast Genome

**Figure 1:**
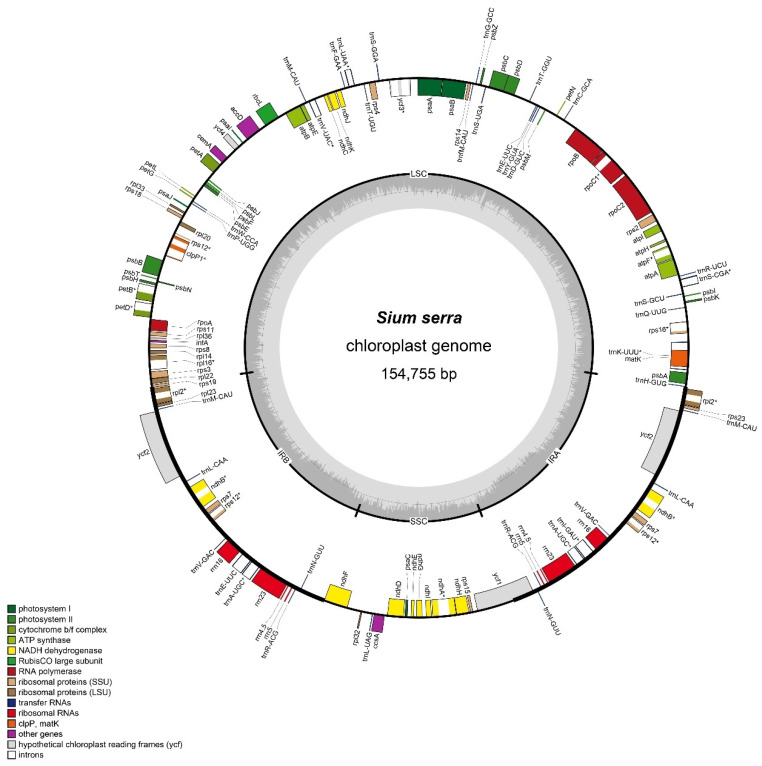
Circular chloroplast (cp) genome map of *Sium serra*. Genes shown outside the large circle are transcribed clockwise, while those inside the circle are transcribed counterclockwise. Genes are color-coded according to their different functional groups. The inner circle represents the GC content. * Gene with one intron.

The chloroplast genome sequence of *S. serra* was 154,755 bp and featured a representative circular structure, including an LSC region of 84,581 bp, an SSC region of 17,664 bp, and a pair of 26,255 bp IR (IRa/IRb) (Figure 1). The genome contained 113 genes, comprising 79 protein-coding, 30 tRNA, and 4 rRNA genes. In addition, the genes duplicated by IR included 6 protein-coding, 7 tRNA, and 4 rRNA genes, resulting in 130 genes. Eighteen genes contained introns, including six tRNA and ten protein-coding genes with a single intron, while *clp*P and *ycf*3 had two introns (Supplementary Table S2). The GC content of the *S. serra* cp genome was 37.4%, consistent with other *Sium* plastomes (Table 1).

### 3.2. Comparative Analysis of Sequence Divergence in the Sium cp Genomes

**Figure 2:**
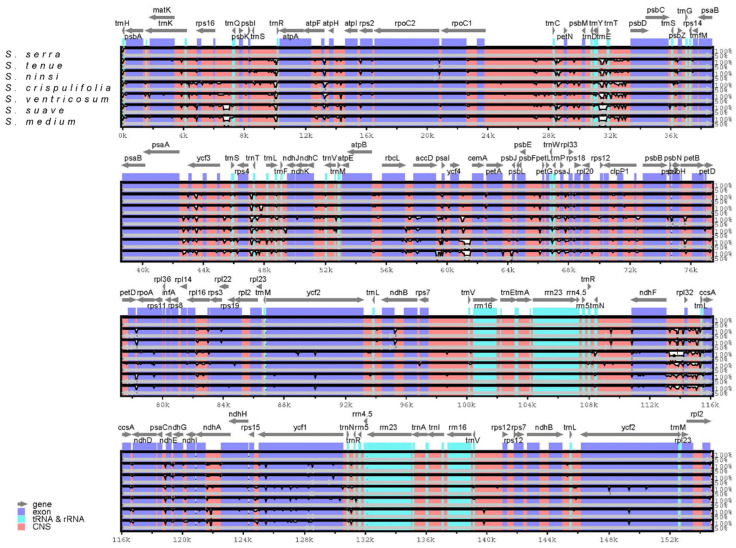
The cp genome sequence alignment of seven *Sium* species, with *S. serra* serving as a reference. Sequence similarities were calculated and visualized using mVISTA.

The sequence variation across the cp genomes of seven *Sium* species was plotted using the mVISTA program, with the annotated *S. serra* cp genome serving as the reference (Figure 2). For the overall sequence identity, as indicated by the peaks and valleys across all seven *Sium* species, the results showed that the LSC and SSC regions were divergent, while the IR region pairs were highly conserved. Three noncoding regions (*rps*16-*trn*Q, *trn*E-*trn*Y, and *ycf*4-*cem*A) in the SC regions exhibited low similarity owing to indel events exceeding 350 bp, which only occurred in the northern Holarctic clade (*S. suave*, *S. medium*).

**Figure 3:**
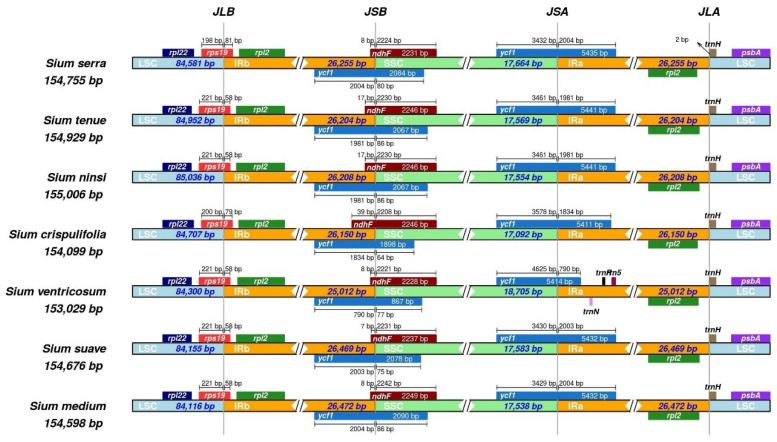
Comparisons of the junctions among the cp genomes of *Sium*. JLB, the junction between the LSC/IRb; JSB, the junction between the SSC/IRb; JSA, the junction between the SSC/IRa; JLA, the junction between the LSC/IRa.

The IR and SC junctions in seven *Sium* species were compared (Figure 3). The longest LSC was observed in *S. ninsi* (85,036 bp), SSC in *S. ventricosum* (18,705 bp), and IR in *S. medium* (26,472 bp). In the cp genomes of all the *Sium* species, *rps*19 was located in JLB, *ndh*F in JSB, and *ycf*1 in JSA. In the case of *S. serra*, unlike the other *Sium* species, *trn*H was located 2 bp away from JLA. In *S. ventricosum*, the *ycf*1 gene present in IRa was shortened as a result of IR contraction and SSC expansion of approximately 1000 bp.

**Figure 4:**
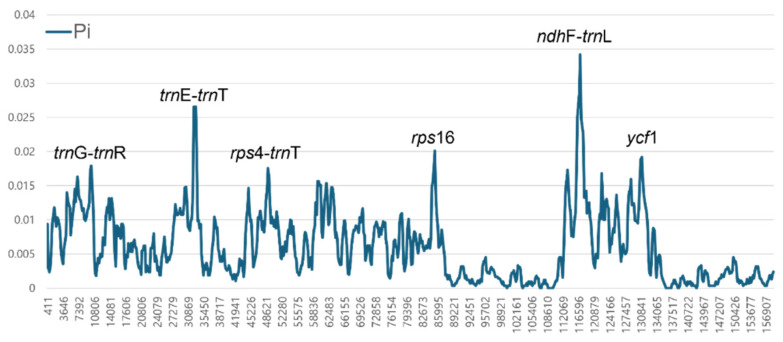
Sliding window analysis of the whole cp genome showing the nucleotide diversity (Pi) compared across seven *Sium* species.

The nucleotide diversity analysis performed with DnaSP showed highly variable regions across the seven *Sium* cp genomes (Figure 4). The mean nucleotide diversity (Pi) across the entire cp genome was 0.00607. Three highly variable regions were identified, each exhibiting a markedly higher Pi value of >0.02. The most variable region was the *ndh*F-*trn*L intergenic spacer in SSC, which had a Pi value of 0.3417. Polymorphism was detected in seven species due to substitution and short-region indels. Other highly variable regions included in *trn*E-*trn*T (Pi = 0.0265) and *rps*16 intron (Pi = 0.0201) within the LSC regions.

### 3.3. Comparative Analysis of the Repeat Sequences in the Sium cp Genomes

**Figure 5:**
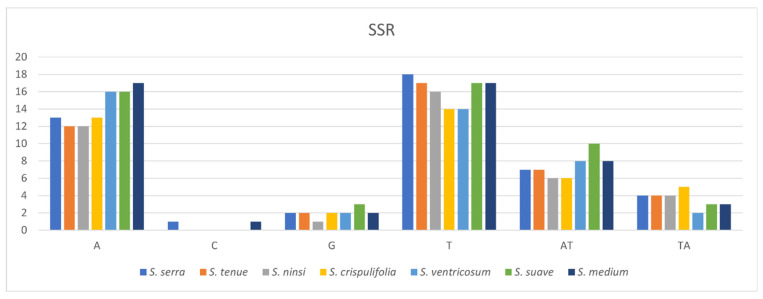
Distribution of SSRs in the *Sium* species.

The results showed that the total number of SSRs ranged from 39 (*S. ninsi*) to 49 (*S. suave*). The distribution of the SSRs varied across the seven cp genomes of *Sium* (Figure 5, Supplementary Table S3). In all the *Sium* species, only two types were found: mononucleotide and dinucleotide. Mononucleotides comprised 74.8%, while dinucleotides accounted for 25.2%. Among the mononucleotides, 93% of the SSRs in all the cp genomes were of the A and T types. For the dinucleotides, only the AT and TA types were observed.

**Figure 6:**
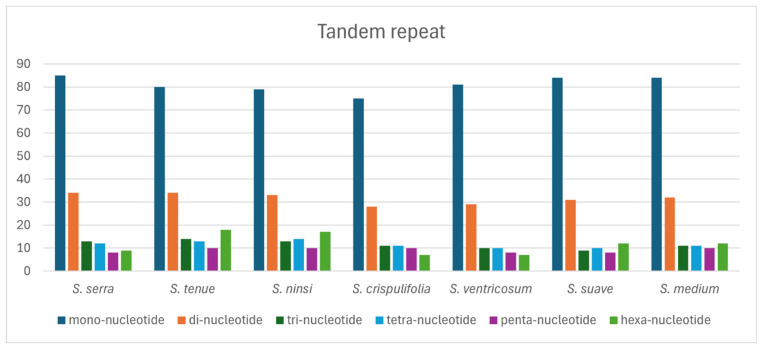
Distribution of tandem repeats in the *Sium* species.

The pattern of tandem repeats in the *Sium* cp genomes ranged from 142 to 169 (Figure 6, Supplementary Table S3). *S. tenue* had the highest number of hexanucleotides, while *S. crispulifolia* and *S. ventricosum* had the lowest.

**Figure 7:**
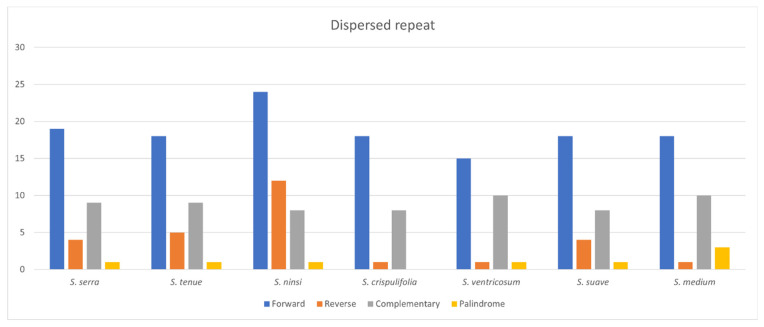
Distribution of dispersed repeats in the *Sium* species.

In the *Sium* cp genomes, the observed repeat types were forward (57%), complementary (27.2%), reverse (12.3%), and palindrome (3.5%). Palindromic repeats were absent from *S. crispulifolia* (Figure 7, Supplementary Table S3).

### 3.4. Adaptive Evolution Analysis in the Sium cp Genomes

**Figure 8:**
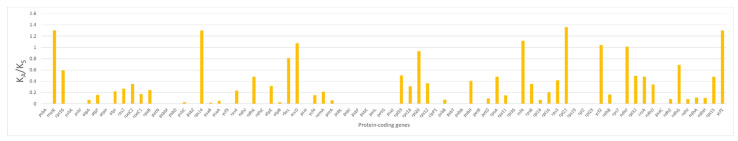
Selective pressure of 79 protein-coding genes across the seven *Sium* species.

Seventy-nine shared protein-coding genes from all seven *Sium* cp genomes were used to calculate the K_S_ and K_A_ substitution rates (Figure 8, Supplementary Table S4). The results showed that most protein-coding genes had a low average K_S_ value (<0.05), except for the *mat*K, *acc*D, *ycf*2, *ndh*F, *rpl*32, and *ycf*1 genes. Similarly, most protein-coding genes exhibited a low average K_A_ value (<0.05), with the exceptions of *mat*K, *acc*D, *rpl*22, *ycf*2, *ndh*F, and *ycf*1 genes. The average K_A_/K_S_ value was 0.26, with a range of K_A_/K_S_ from 0 to 1.35 (Figure 8). Eight genes (*mat*K, *rps*14, *acc*D, *inf*A, *rpl*22, *ycf*2, *ndh*F, and *ycf*1) exhibited high average K_A_/K_S_ values (>1). Among these, *mat*K, *acc*D, *ycf*2, *ndh*F, and *ycf*1 genes had K_A_ and K_S_ values that were also higher than those of other genes.

### 3.5. Phylogenetic Analysis of the Genus Sium

**Figure 9:**
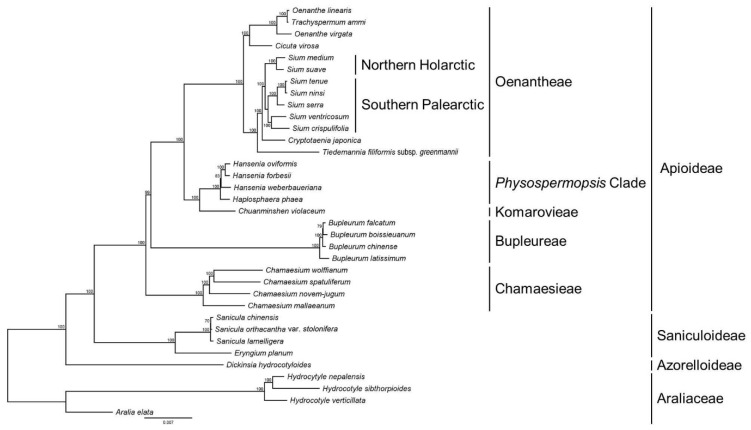
Maximum likelihood (ML) tree for 35 taxa based on 79 common plastid protein-coding genes. Values above the branches represent the ML bootstrap value. Abbreviation: maximum likelihood, ML.

Phylogenetic analysis was performed using 79 protein-coding genes from the *Sium* and related Apioideae taxa (Figure 9). The genus *Sium* was found to be monophyletic, clustering into two lineages (Southern Palearctic and Northern Holarctic Clade). The Southern Palearctic clade included *S. crispulifolia*, *S. ventricosum*, *S. serra*, *S. ninsi*, and *S. tenue*, which are biogeographically native to the southern Palearctic. The Northern Holarctic clade included *S. suave* and *S. medium*, which are species native to the Northern Holarctic region. All the *Sium* species were supported with strong bootstrap values (BS = 100). These findings indicate that *Sium* forms a sister group to *Cryptotaenia*.

## 4. Discussion

In this study, we sequenced and analyzed *Sium serra* (Franch. & Sav.) Kitag. distributed in Korea, China, and Japan. Compared to previous studies, the length and gene contents of the cp genome in the genus *Sium* were conserved [34]. Events such as gene loss or rearrangement did not occur in the genus *Sium*. However, some noncoding regions in the SC exhibited low similarity, which could result from indel events (Figure 2). The total cp genome size of *Sium* species varied between 153 kb in *S. ventricosum* and 155 kb in *S. ninsi*. Among the seven *Sium* cp genomes, *S. serra* exhibited the third-largest genome size (Figure 3). The average size of *Sium* cp genomes is 154.4 kb, similar to those of previously published Apioideae cp genomes [35,36]. Among the seven *Sium* cp genomes, *S. ventricosum* had the smallest size, which could result from an IR contraction and SSC expansion of approximately 1000 bp. This indicates that the *ycf*1 gene at the SSC/IRa junction (JSA) is predominantly located in the SSC (Figure 3). A previous study reported that the *ndh*F gene is entirely located in the SSC region for three taxa (*S. ninsi*, *S. tenus*, and *S. ventricosum*) [34]. However, in this study, the *ndh*F gene was found to be located at the SSC/IRb junction (JSB) in all the taxa. This discrepancy is likely due to an error in the previous search. Furthermore, the Southern Palearctic clade exhibited a shorter IR region compared to the Northern Holarctic clade, likely due to IR contraction. In addition, the clade containing *S. serra* showed a slightly larger IR region than those of the other Southern Palearctic clades.

Consequently, nucleotide diversity analysis revealed approximately six highly variable sites across seven *Sium* species (Figure 4). This site is located in the SC, indicating that the IR regions are more conservative. Additionally, this finding is almost similar to that of a previous study [34]. The highest nucleotide diversity (Pi = 0.03417) is present in the *ndh*F-*trn*L region, which exhibits significant variation. These highly variable sites could function as effective molecular markers for shallow-level taxonomic studies between and within specific taxa of the genus *Sium* [37,38].

According to our knowledge, we analyzed the repeat sequences in the plastomes of the genus *Sium* for the first time. Repeat sequences play a crucial role in homologous recombination in plant genomes [39]. The distribution of the SSRs in the cp genome of *Sium* ranged from 39 (*S. ninsi*) to 49 (*S. suave*) (Figure 5, Supplementary Table S3). Most SSRs are mononucleotides, comprising 74.8%, while the others are dinucleotides. The C repeat sequence is only present in *S. serra* and *S. medium*. The distribution of tandem repeats in the plastomes of *Sium* ranged from 142 (*S. crispulifolia*) to 169 (*S. tenue*) (Figure 6, Supplementary Table S3). In this study, *S. tenue* had the highest number of hexanucleotide tandem repeats in the genus *Sium*. The distribution of dispersed repeats in the plastomes of *Sium* ranged from 27 (*S. crispulifolia*, *S. ventricosum*) to 45 (*S. ninsi*) (Figure 7, Supplementary Table S3). No palindrome-type repeats were present only in *S. crispulifolia*, *S. tenue,* and *S. ninsi.* These species are primarily distinguished by differences in the leaf width [4]. This repeat sequence may serve as a powerful species identification marker within the genus *Sium* [40,41]. Furthermore, it can help distinguish *Sium* species from Gao-ben, which is used as an herbal medicine [8].

Vascular plants adapt to various ecological niches. Factors such as the light intensity, moisture levels, and temperature influence their photosynthesis. Owing to adaptation to these factors, genes undergo selection [42]. Several genes undergo positive selection in this study (Figure 8, Supplementary Table S4). The *mat*K gene is involved in group Ⅱ intron splicing. This gene is also positioned in the intron of *trn*K and exhibits a high substitution rate [43]. In this study, the K_A_/K_S_ ratio of the *mat*K gene was confirmed to be 1.298, indicating positive selection. The *rpl*22 gene encodes the chloroplast ribosomal protein CL22, which is associated with the large ribosomal protein subunit [44]. In the genus *Sium*, the K_A_ and K_S_ values of the *rpl*22 gene were approximately 0.5, while the K_A_/K_S_ ratio was the highest at 1.356. This result could be attributed to the fact that most *Sium* species grow in shaded, humid environments. In addition, a similar pattern is also observed in the genus *Chrysosplenium*, which grows in a similar environment [45]. Furthermore, genes subjected to positive selection are distributed across the LSC and SSC regions, showing a similar trend to that observed in the nucleotide diversity.

Phylogenetic studies of the genus *Sium* have been conducted in the past. Analyses using nuclear rDNA ITS and a few cp DNAs reveal that *Sium* does not form a monophyletic group but is polyphyletic with the genus *Berula* [46,47,48]. In addition, a study using the cp genome was also conducted; however, *S. serra* was excluded because it is classified as *Pimpinella* in China [5,34]. In this study, all the species within the genus *Sium*, including *S. serra*, form a single clade with high bootstrap values. Moreover, as in previous studies, two subclades are formed, supporting the results of biogeographic studies on the genus *Sium* [2,34].

## 5. Conclusions

In this study, the chloroplast genome of *S. serra* was sequenced and assembled for the first time. The detailed characteristics of the *S. serra* chloroplast genome were identified through an analysis of its features. All the *Sium* species have typical circular structures, are 153–155 kb long, and contain 130 genes. Hotspots and repeat sequences such as SSRs and tandem repeats can be used as molecular markers at the intraspecific level. The substitution rates analyzed in this study can be applied to other plants living in similar environments. Finally, the genus *Sium* formed a monophyletic group. Furthermore, these genetic resources serve as a reference for further molecular phylogenetic studies on the Oenantheae tribe and, more generally, the Apiaceae family.

## Figures and Tables

**Table 1:** Characteristic features of the *S*. *serra* chloroplast genome.

Sequence Region		*S. serra*
Total chloroplast genome size (bp)		154,755
LSC length (bp)		84,581
SSC length (bp)		17,664
IR length (bp)		26,255
Total number of genes		130
Protein-coding genes		79
tRNA genes		30
rRNA genes		4
Gene duplicated by IR		17
Genes with introns		18
GC content	Total (%)	37.4
	LSC (%)	35.5
	SSC (%)	30.7
	IR (%)	42.7
	CDS (%)	37.9
	tRNA (%)	52.9
	rRNA (%)	55.3
Protein-coding genes (% bp)		50.27
All genes (% bp)		57.94

## Data Availability

The sequence data generated in this study are available in GenBank of the National Center for Biotechnology Information (NCBI) under the access number PP941959.

## Supplementary Materials

The following supporting information can be downloaded at: https://www.mdpi.com/article/10.3390/genes15121567/s1. Table S1. List of taxa and GenBank accession numbers used in the phylogenetic analysis. Table S2. List of genes present in the chloroplast genom of *S. serra.* Table S3. Distribution of distinct type of repeats in the 7 *Sium* cp genomes. Table S4. Amount of synonymous and non-synonymous substitution rates present in the 79 protein-coding genes of the 7 *Sium* cp genomes.
