# Supplementary material for: Comparative and Adaptive Analyses of the Complete Chloroplast Genome Diversity in Sium serra
Source: Genes (Basel). 2024 Dec 3;15(12):1567. doi: 10.3390/genes15121567 (PMC11728278; doi:10.3390/genes15121567)
Supplement: Supplementary file 1 [file genes-15-01567-s001.zip › Supplementary Table S1.pdf]

Supplementary Table S1. List of taxa and GenBank accession numbers used in the phylogenetic analysis.

S. N.	Species name	NCBI number
1.	*Oenanthe linearis*	MT561035
2.	*Trachyspermum ammi*	NC_047246
3.	*Oenanthe virgata*	KX832335.1
4.	*Cicuta virosa*	NC_037711
5.	*Cryptotaenia japonica*	NC_046737
6.	*Sium suave*	NC_071929
7.	*Sium medium*	NC_072108
8.	*Sium tenue*	NC_072106
9.	*Sium ninsi*	NC_072107
10.	*Sium serra*	PP941959 (In this study)
11.	*Sium ventricosum*	OP234514
12.	*Sium crispulifolium*	NC_072109
13.	*Tiedemannia filiformis* subsp. *Greenmannii*	HM596071
14.	*Hansenia oviformis*	MG197730
15.	*Hansenia forbesii*	MW820162
16.	*Hansenia weberbaueirana*	MW820163
17.	*Haplosphaera phaea*	MK801097
18.	*Chuanminshen violaceum*	KU921430
19.	*Bupleurum boissieuanum*	NC_036017
20.	*Bupleurum falcatum*	NC_027834
21.	*Bupleurum chinense*	MN854378
22.	*Bupleurum latissimum*	NC_033346
23.	*Chamaesium wolffianum*	MN119374
24.	*Chamaesium spatuliferum*	MN119371
25.	*Chamaesium novem-jugum*	MN119370
26.	*Chamaesium mallaeanum*	MN119369
27.	*Sanicula lamelligera*	OP703174
28.	*Sanicula chinensis*	MK208987
29.	*Sanicula orthacantha* var. *stolonifera*	MT561028
30.	*Eryngium planum*	MT561039
31.	*Dickinsia hydrocotyloides*	MT423729
32.	*Hydrocotyle nepalensis*	MT561038
33.	*Hydrocotyle sibthorpioides*	NC_035502
34.	*Hydrocotyle verticillata*	NC_015818
35.	*Aralia elata*	KT153023
