# Supplementary material for: Comparative and Adaptive Analyses of the Complete Chloroplast Genome Diversity in Sium serra
Source: Genes (Basel). 2024 Dec 3;15(12):1567. doi: 10.3390/genes15121567 (PMC11728278; doi:10.3390/genes15121567)
Supplement: Supplementary file 1 [file genes-15-01567-s001.zip › Supplementary Table S2.pdf]

Supplementary Table S2. List of genes present in the chloroplast genome of *S. serra*.

Category of Genes	Group of Genes	Gene Name
Photosynthesis-related genes	Large subunit of rubisco	*rbc*L
	Photosystem Ⅰ	*psa*A, *psa*B, *psa*C, *psa*I, *psa*J
	Assembly / stability of photosystem Ⅰ	y*cf*3**, *ycf*4
	Photosystem Ⅱ	*psb*A, *psb*B, *psb*C, *psb*E, *psb*F, *psb*H, *psb*I, *psb*J, *psb*K, *psb*L, *psb*M, *psb*N, *psb*T, *psbZ*
	Cytochrome b6/f complex	*pet*A, *pet*B*, *pet*D*, *pet*G, *pet*L, *pet*N
	Cytochrome c synthesis	*ccs*A
	NADH dehydrogenase	*ndh*A*, *ndh*B*(*2), *ndh*C, *ndh*D, *ndh*E, *ndh*F, *ndh*G, *ndh*H, *ndh*I, *ndh*J, *ndh*K
Transcription and translation related genes	RNA polymerase subunits / transcription	*rpo*A, *rpo*B, *rpo*C1*, *rpo*C2
	Small subunit of ribosomal proteins	*rps*2, *rps*3, *rps*4, *rps*7(*2), *rps*8, *rps*11, *rps*12**(*2), *rps*14, *rps*15, *rps*16*, *rps*18, *rps*19
	Large subunit of ribosomal proteins	*rpl*2*(*2), *rpl*14, *rpl*16*, *rpl*20, *rpl*22, *rpl*23(*2), *rpl32, rpl*33, *rpl*36
	Translation initiation factor	*inf*A
RNA genes	Ribosomal RNA	*rrn*4.5(*2), *rrn*5(*2), *rrn*16(*2), *rrn*23(*2)
	Transfer RNA	*trn*A-UGC*(*2), *trn*C-GCA, *trn*D-GUC, *trn*E-UUC, *trn*F-GAA, *trn*G-GCC, *trn*G-UCC*, *trn*H-GUG, *trn*I-CAU(*2), *trn*I-GAU*(*2), *trn*K-UUU*, *trn*L-CAA(*2), *trn*L-UAA*, *trn*L-UAG, *trnf*M-CAU, *trn*M-CAU, *trn*N-GUU(*2), *trn*P-UGG, *trn*Q-UUG, *trn*R-ACG(*2), *trn*R-UCU, *trn*S-GCU, *trn*S-GGA, *trn*S-UGA, *trn*T-GGU, *trn*T-UGU, *trn*V-GAC(*2), *trn*V-UAC*, *trn*W-CCA, *trn*I-GAU*(*2)
Other genes	RNA processing	*mat*K
	Carbon metabolism	*cem*A
	Fatty acid synthesis	*acc*D
	Proteolysis	*clp*P**
	Component of TIC complex	*ycf*1
	Hypothetical proteins	*ycf*2(*2)

* Gene with one intron, ** Gene with two introns, (*2) Gene with two copies.
